# The Effects of Using Aluminum Oxide Nanoparticles as Heat Transfer Fillers on Morphology and Thermal Performances of Form-Stable Phase Change Fibrous Membranes Based on Capric–Palmitic–Stearic Acid Ternary Eutectic/Polyacrylonitrile Composite

**DOI:** 10.3390/ma11091785

**Published:** 2018-09-19

**Authors:** Huizhen Ke, Yonggui Li

**Affiliations:** Fujian Key Laboratory of Novel Functional Textile Fibers and Materials, Faculty of Clothing and Design, Minjiang University, Fuzhou 350108, Fujian, China; liyonggui2018@mju.edu.cn

**Keywords:** physical adsorption, aluminum oxide, phase change composite fibrous membrane, thermal energy storage, melting and freezing times

## Abstract

In this paper, innovative capric–palmitic–stearic acid ternary eutectic/polyacrylonitrile/aluminum oxide (CA–PA–SA/PAN/Al_2_O_3_) form-stable phase change composite fibrous membranes (PCCFMs) with different mass ratios of Al_2_O_3_ nanoparticles were prepared for thermal energy storage. The influences of Al_2_O_3_ nanoparticles on morphology and thermal performances of the form-stable PCCFMs were investigated by scanning electron microscopy (SEM), differential scanning calorimetry (DSC), and measurement of melting and freezing times, respectively. The results showed that there was no apparent leakage trace from the SEM observation. The DSC analysis indicated that the addition of Al_2_O_3_ nanoparticles had no significant effect on phase transition temperatures and enthalpies of the CA–PA–SA/PAN/Al_2_O_3_ form-stable PCCFMs. The melting peak temperatures and melting enthalpies of form-stable PCCFMs were about 25 °C and 131–139 kJ/kg, respectively. The melting and freezing times of the CA–PA–SA/PAN/Al_2_O_3_10 form-stable PCCFMs were shortened by approximately 21% and 23%, respectively, compared with those of the CA–PA–SA/PAN form-stable PCCFMs due to the addition of Al_2_O_3_ nanoparticles acting as heat transfer fillers.

## 1. Introduction

Thermal energy storage technologies for reducing energy consumption and improving energy efficiency have become increasingly important with the growth of population and the development of industry. Among the available strategies for energy storage, latent heat storage technology using phase change materials (PCMs) is considered as one of the most promising technologies. Currently, PCMs have been widely researched and successfully applied in numerous fields such as solar thermal collectors [1], heat recovery systems [2], residential and commercial buildings [3], refrigerated containers [4], greenhouse systems [5], Li-ion battery thermal management [6], thermal regulating fibers and textiles [7], and so forth.

Fatty acid eutectics, as a derivative of fatty acids, exhibit the same desirable properties as those of fatty acids, such as good thermal and chemical stabilities, high latent heat storage capacities, reversible phase change behavior, no supercooling and phase segregation, nontoxicity, noncorrosiveness, and cost effectiveness [8]. However, low thermal conductivity and liquid leakage problems limit their feasibility in thermal energy storage applications to a certain extent. According to the literature, the disadvantage of liquid leakage of organic solid–liquid PCMs can be overcome by combining them with supporting materials to develop form-stable PCMs through different methods such as absorbing method [9,10], miniemulsion polymerization [11], melt mixing method [12], vacuum impregnation method [13,14], electrospinning [15,16,17], casting molding method [18], physical adsorption [19,20], emulsion evaporation method [21], and so on. The supporting materials involve various materials including inorganic materials (e.g., expanded perlite [9], diatomite [10], expanded vermiculite [13]) and polymers (e.g., poly(methyl methacrylate) [11], linear low-density polyethylene [12], ethylene-vinyl acetate [14], polyacrylonitrile [15], polyamide 6 [16], polyethylene terephthalate [17], epoxy resin [18], polyurethane [19], cellulose acetate [20], polylactic acid [21]). Moreover, the overall heat transfer efficiencies of phase transition systems can be improved by adding or dispersing heat transfer fillers with high thermal conductivity such as metal materials (e.g., silver nanoparticles [19] and copper foam [22]), carbon materials (e.g., expanded graphite [10,12], carbon fibers [14], carbon nanotubes [23], graphene oxide, and graphene nanoplatelets [24]), and ceramic materials (e.g., hexagonal boron nitride [25] and aluminium oxide nanoparticles [26]). Among the above-mentioned materials, aluminium oxide (Al_2_O_3_) is a promising ceramic thermal conductivity enhancer due to its outstanding advantages, such as stable chemical property, high thermal conductivity (about 30 W/(m.K)), good dispersity within the solid–liquid PCMs, and greater cost-effectiveness compared to other ceramic materials (e.g., boron nitride).

According to the literature, there is no report about the preparation and investigation of electrospun polymer-based nanofibrous membranes loading different amounts of Al_2_O_3_ nanoparticles acting as supporting materials. Therefore, the objective of this paper was to prepare a new kind of supporting material to develop form-stable PCMs in which electrospun polyacrylonitrile (PAN) nanofibrous membranes acted as polymer supporting matrices and the Al_2_O_3_ nanoparticles acted as heat transfer fillers. Moreover, the capric–palmitic–stearic acid ternary eutectic (CA–PA–SA) with the melting peak temperature of about 25 °C was prepared as solid–liquid PCMs. Thereafter, the innovative CA–PA–SA/PAN/Al_2_O_3_ form-stable phase change composite fibrous membranes (PCCFMs) with different mass fractions of Al_2_O_3_ nanoparticles (i.e., 0, 5, and 10 wt.%) were fabricated by physical adsorption. The morphological structure, phase change temperatures, and enthalpies, as well as thermal energy storage and release performances of the CA–PA–SA/PAN/Al_2_O_3_ form-stable PCCFMs were systematically studied by scanning electron microscopy (SEM), differential scanning calorimetry (DSC), and measurement of melting and freezing times, respectively.

## 2. Experimental

### 2.1. Materials

The polyacrylonitrile (PAN, M_w_ = 150,000) powder was purchased from Polysciences, Inc. (Warrington, PA, USA) The aluminum oxide (Al_2_O_3_) nanoparticles with >99 wt.% purity and 8–12 nm particle diameter were supplied by Nanjing XFNANO Materials Tech Co., Ltd. (Nanjing, China). The chemicals, including capric acid (CA), palmitic acid (PA), stearic acid (SA), and N,N-dimethyl formamide (DMF) were purchased from Sinopharm Group Chemical Reagent Co., Ltd. (Shanghai, China). All of the chemicals were used as received without further purification.

### 2.2. Fabrication of Electrospun PAN/Al_2_O_3_ Supporting Membranes

In this paper, the PAN/Al_2_O_3_ supporting membranes with different amounts of Al_2_O_3_ nanoparticles were fabricated by electrospinning. The 10 wt.% PAN solution was firstly prepared by dissolving the PAN powder into the DMF solvent. Thereafter, the Al_2_O_3_ nanoparticles were added into the PAN solution with the mass ratios [W_Al_2_O_3__: (W_PAN_ + W_Al_2_O_3__)] of 5 wt.% and 10 wt.%. Finally, these PAN/Al_2_O_3_ composite solutions were magnetically stirred to achieve the homogeneous solutions for electrospinning. The equipment for the electrospinning process was comprised of high-voltage power supply, syringe pump, and collector. The PAN or PAN/Al_2_O_3_ solution was placed into a 20 mL syringe with a stainless-steel needle whose inner diameter was 0.6 mm. The solution feed rate was set at 1 mL/h. A roller wrapped with aluminum foil was used as the collector, which was connected to the ground. A positive high voltage was applied to the needle during the electrospinning process. The electrospinning parameters, such as applied voltage, tip-to-collector distance, and rotating speed of the roller, were fixed at 18 kV, 18 cm, and 200 rpm, respectively. The obtained nanofibrous membranes were named as PAN, PAN/Al_2_O_3_5, and PAN/Al_2_O_3_10.

### 2.3. Fabrication of CA–PA–SA/PAN/Al_2_O_3_ Form-Stable PCCFMs

The CA–PA–SA ternary eutectic with eutectic mass ratio of 83.82/10.19/5.99 was prepared as solid–liquid PCMs by heating-ultrasonic method, which has been reported by my previous published literatures [27,28]. The prepared PAN/Al_2_O_3_ supporting membranes were immersed into the molten CA–PA–SA ternary eutectic for 1 h in the oven at 40 °C until the absorption was saturated. Subsequently, the PAN/Al_2_O_3_ nanofibrous membranes absorbed with CA–PA–SA ternary eutectic were hung in the oven for 5 h to removal residual CA–PA–SA ternary eutectic on the surface of membranes. Finally, the prepared form-stable PCCFMs were termed as CA–PA–SA/PAN, CA–PA–SA/PAN/Al_2_O_3_5, and CA–PA–SA/PAN/Al_2_O_3_10. The similar preparation method has been reported in the literatures published by my research group [19,20,28].

### 2.4. Characterizations

#### 2.4.1. Scanning Electron Microscopy

Morphological structure of electrospun PAN and PAN/Al_2_O_3_ nanofibrous membranes, as well as the CA–PA–SA/PAN/Al_2_O_3_ form-stable PCCFMs with different amounts of Al_2_O_3_ nanoparticles, were observed by scanning electron microscope (SEM, S-3400N, Hitachi, Tokyo, Japan).

#### 2.4.2. Differential Scanning Calorimeter

Differential scanning calorimeter (DSC-Q200, TA Instruments-Waters LLC, Shanghai, China was used to investigate the thermal energy storage properties including peak onset temperatures (*T_o_*), melting peak temperatures (*T_m_*), freezing peak temperatures (*T_f_*), peak end temperatures (*T_e_*), melting enthalpies (*ΔH_m_*), and freezing enthalpies (*ΔH_f_*) of the CA–PA–SA ternary eutectic and the CA–PA–SA/PAN/Al_2_O_3_ form-stable PCCFMs from −20 °C to 60 °C with the scanning rate of 8 °C/min under a nitrogen atmosphere. The flow rate of nitrogen was set at 50 mL/min for DSC measurement.

Moreover, good thermal stability over a number of thermal cycles are necessary for form-stable PCCFMs. Therefore, the accelerated thermal cycling experiment containing 100 heating and cooling thermal cycles was conducted to investigate the thermal stability of the CA–PA–SA/PAN/Al_2_O_3_10 form-stable PCCFMs in a climatic chamber (Binder MK56, BINDER GmbH, Tuttlingen, Germany) with the heating and cooling rates of 5 °C/min in the temperature range of −10–50 °C. Subsequently, the phase change temperatures and enthalpies of the CA–PA–SA/PAN/Al_2_O_3_10 form-stable PCCFMs after thermal cycling were also measured by DSC analysis under the same test conditions.

#### 2.4.3. Measurement of Melting and Freezing Times

Improvement of heat transfer performances of the fabricated CA–PA–SA/PAN/Al_2_O_3_ form-stable PCCFMs was evaluated by measurement of melting and freezing times. The CA–PA–SA/PAN/Al_2_O_3_ form-stable PCCFMs were cut and put in the test bottle. The test bottle was placed into a water bath at 40 °C for melting measurement. Thereafter, the test bottle was rapidly transferred to a refrigerator at −10 °C for freezing measurement. A thermocouple positioned at the center of the bottle was employed to measure temperature variation of samples during the testing process. The temperature variation caused by thermal energy storage and release was automatically recorded by a computer with the temperature measuring accuracy of ±2 °C. The same analytical method has also been reported by my previous literatures [19,20,28].

## 3. Results and Discussion

### 3.1. Morphological Structure

**Figure 1:**
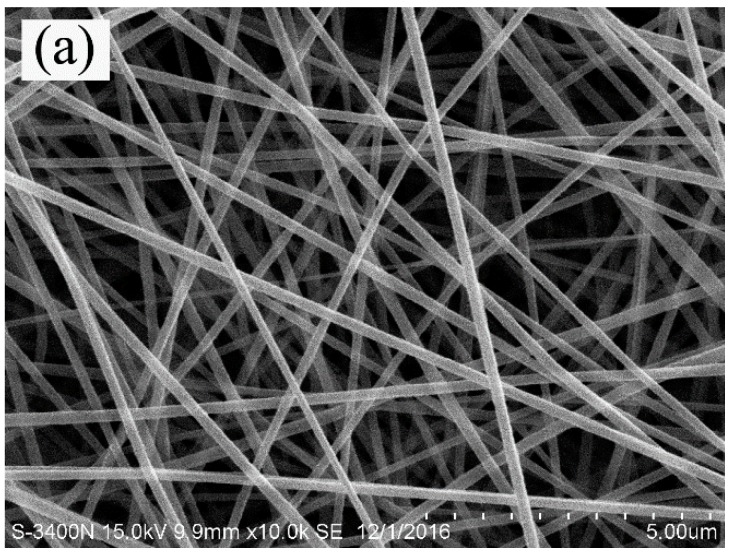

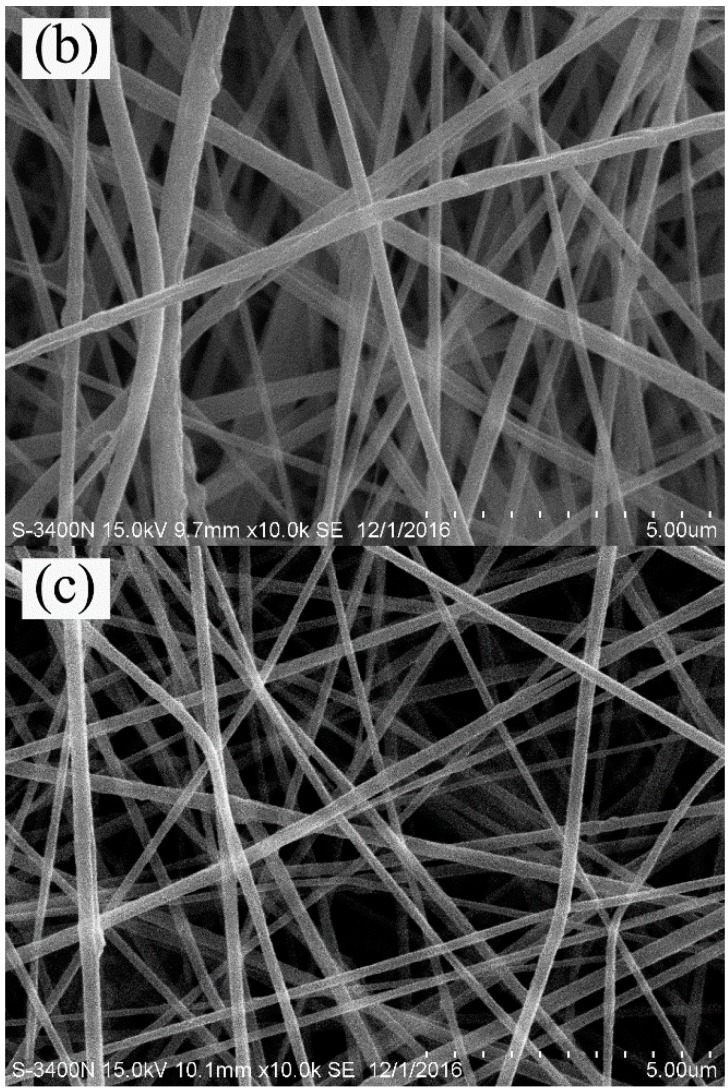
Representative SEM images of electrospun nanofibrous membranes: (**a**) PAN, (**b**) PAN/Al_2_O_3_5, (**c**) PAN/Al_2_O_3_10.

**Figure 2:**
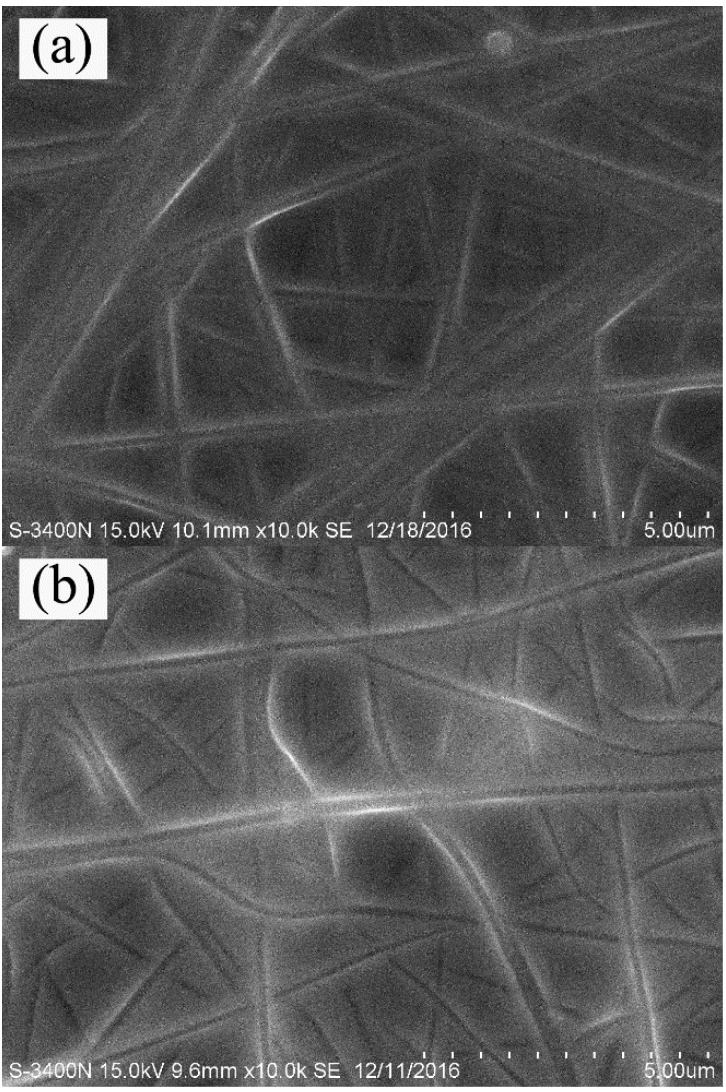

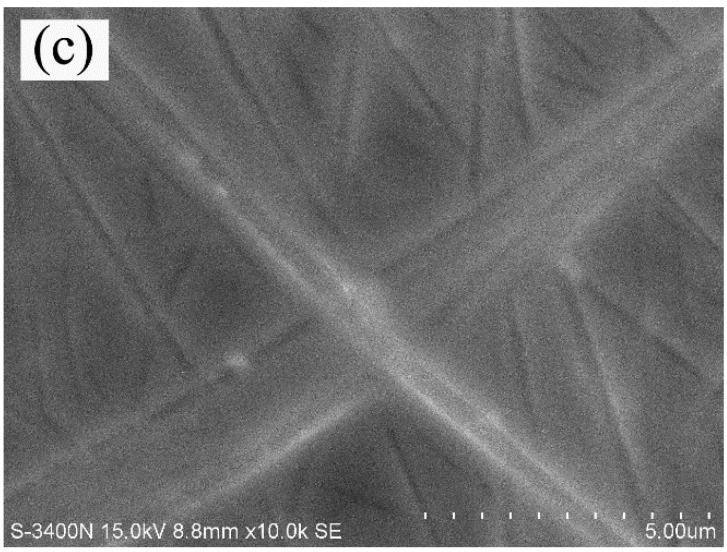
Representative SEM images of form-stable PCCFMs: (**a**) CA–PA–SA/PAN, (**b**) CA–PA–SA/PAN/Al_2_O_3_5, (**c**) CA–PA–SA/PAN/Al_2_O_3_10.

Figure 1 shows the SEM images of electrospun PAN fibrous membrane, PAN/Al_2_O_3_5, and PAN/Al_2_O_3_10 composite fibrous membranes. As shown in Figure 1, electrospun PAN nanofibers exhibited the smooth surface and uniform fiber diameters along their lengths. The average fiber diameter was observed to be about 200 nm. It can be clearly seen from Figure 1 that electrospun PAN nanofibers were randomly deposited to form fibrous membranes with a three-dimensional porous network structure during the electrospinning process. Figure 1b,c reveal that electrospun PAN/Al_2_O_3_ composite fibrous membranes with different amounts of Al_2_O_3_ nanoparticles also presented three-dimensional porous network structures consisting of randomly distributed PAN/Al_2_O_3_ composite nanofibers with the average fiber diameter being in the range of approximately 218–274 nm. It was clear that electrospun PAN/Al_2_O_3_ composite nanofibers showed a relatively rough surface structure compared to that of the pure PAN nanofibers owing to the addition of Al_2_O_3_ nanoparticles. Moreover, Figure 2 presents the SEM images of the CA–PA–SA/PAN, CA–PA–SA/PAN/Al_2_O_3_5, and CA–PA–SA/PAN/Al_2_O_3_10 form-stable PCCFMs. It can be clearly found from Figure 2 that the CA–PA–SA ternary eutectic was uniformly adsorbed into the three-dimensional porous network structure of electrospun PAN/Al_2_O_3_ fibrous membranes due to the high infiltrating ability of the CA–PA–SA ternary eutectic as well as capillary effect and surface tension force of fibrous membranes. Figure 2 indicated that the combination of Al_2_O_3_ nanoparticles had no significant effect on the morphological structure of the CA–PA–SA/PAN/Al_2_O_3_ form-stable PCCFMs. It is worthwhile to note that the melting temperature of the CA–PA–SA ternary eutectic was in the range of around 20–33 °C, and its melting peak temperature was about 25 °C (see Table 1). As we know, the working temperature of SEM characterization was also about 25 °C, which meant that the solid–liquid phase change behavior (i.e., melting process) of the CA–PA–SA ternary eutectic adsorbed into the form-stable PCCFMs was taking place during the process of SEM observation. However, the SEM images clearly demonstrated that the melted CA–PA–SA ternary eutectic was still encapsulated into the three-dimensional porous network structure of electrospun PAN/Al_2_O_3_ fibrous membranes even when the ambient temperature was consistent with its melting peak temperature. In other words, the developed CA–PA–SA/PAN/Al_2_O_3_ PCCFMs exhibited a form-stable structure because of the supporting effect of electrospun PAN/Al_2_O_3_ nanofibrous membranes.

### 3.2. Thermal Energy Storage Properties

**Figure 3:**
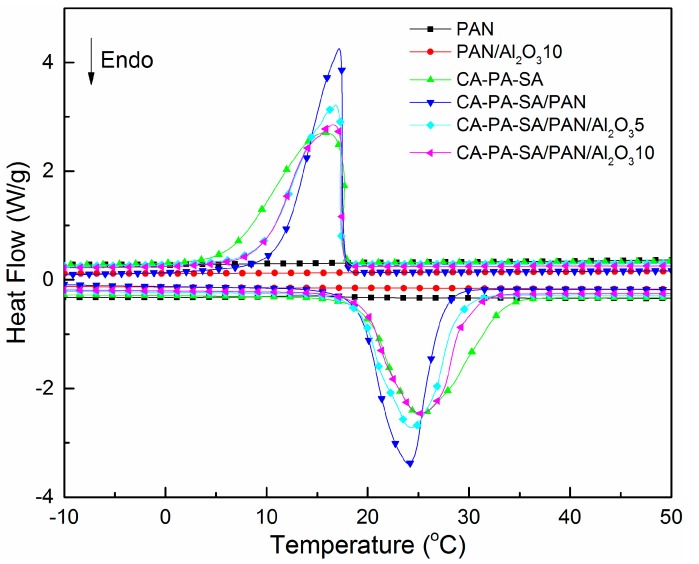
DSC curves of the CA–PA–SA ternary eutectic, electrospun PAN, and PAN/Al_2_O_3_10 nanofibrous membranes, as well as the CA–PA–SA/PAN/Al_2_O_3_ form-stable PCCFMs with different amounts of Al_2_O_3_ nanoparticles.

Figure 3 illustrates DSC curves of the CA–PA–SA ternary eutectic and the CA–PA–SA/PAN/Al_2_O_3_ form-stable PCCFMs with different amounts of Al_2_O_3_ nanoparticles during melting and freezing processes. The obtained thermal performance data are listed in Table 1. It can be clearly seen from Figure 3 that no solid–liquid phase change peak (i.e., endothermic or exothermic peak) was observed from the DSC curves of electrospun PAN and PAN/Al_2_O_3_10 nanofibrous membranes, which confirmed that electrospun PAN and PAN/Al_2_O_3_ nanofibrous membranes acting as supporting materials did not provide any phase change enthalpies within the temperature range of the DSC measurement. As shown in Table 1, the melting temperatures of the CA–PA–SA ternary eutectic were in the range of about 20–33 °C, and its melting peak temperature was approximately 25 °C. It is interesting to note that this temperature was generally considered as the most appropriate temperature for temperature regulation fibers and textiles as well as energy-saving building applications, thus the CA–PA–SA ternary eutectic was chosen as solid–liquid PCMs in this paper. Figure 3 presents that only one endothermic or exothermic peak was observed from the DSC curves of the CA–PA–SA/PAN/Al_2_O_3_ form-stable PCCFMs, which was similar to those of the pure CA–PA–SA ternary eutectic. DSC analysis also indicated that the phase change temperatures of the CA–PA–SA/PAN/Al_2_O_3_ form-stable PCCFMs were around 10–30 °C, as revealed in Table 1. Additionally, compared with those of pure CA–PA–SA ternary eutectic, the phase change enthalpies of the CA–PA–SA/PAN/Al_2_O_3_ form-stable PCCFMs had a varying degree of reduction because electrospun PAN/Al_2_O_3_ nanofibrous membranes acting as supporting materials accounted for a certain proportion of the form-stable PCCFMs. Meanwhile, Figure 3 and Table 1 also suggest that the addition of Al_2_O_3_ nanoparticles had no significant effect on the phase transition temperatures and enthalpies of the CA–PA–SA/PAN/Al_2_O_3_ form-stable PCCFMs in comparison to those of the CA–PA–SA/PAN form-stable PCCFMs. The absorption capacities of electrospun PAN/Al_2_O_3_ nanofibrous membranes with different amounts of Al_2_O_3_ nanoparticles on the CA–PA–SA ternary eutectic were also determined in this paper (i.e., melting enthalpy of the CA–PA–SA/PAN/Al_2_O_3_ form-stable PCCFMs divided by melting enthalpy of the CA–PA–SA ternary eutectic). The corresponding absorption capacities were respectively confirmed as 95.13% for CA–PA–SA/PAN, 90.80% for CA–PA–SA/PAN/Al_2_O_3_5, and 89.98% for CA–PA–SA/PAN/Al_2_O_3_10, indicating that electrospun PAN and PAN/Al_2_O_3_ nanofibrous membranes had high absorption capacity on the CA–PA–SA ternary eutectic. This might have resulted because the excellent features of nanofibrous membranes, such as porous network structure, as well as the high surface-to-volume ratio, had a positive contribution to absorbing large amounts of solid–liquid PCMs.

**Figure 4:**
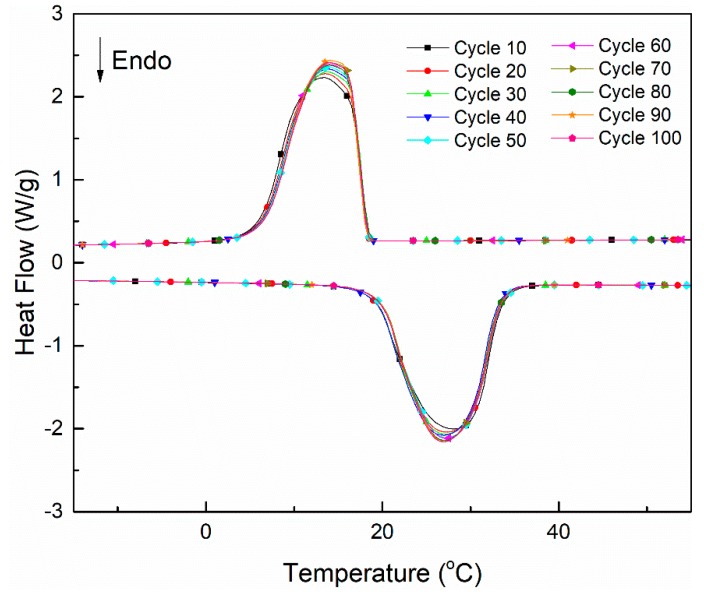
DSC curves of the CA–PA–SA/PAN/Al_2_O_3_10 form-stable PCCFMs after thermal cycles.

Figure 4 shows the DSC curves of the CA–PA–SA/PAN/Al_2_O_3_10 form-stable PCCFMs after thermal cycling. The phase change temperatures and enthalpies extracted from DSC curves are summarized in Table 2. It can be seen from Figure 4 that the endothermic and exothermic curves of the CA–PA–SA/PAN/Al_2_O_3_10 form-stable PCCFMs after thermal cycling had no obvious variation compared with each other. It should be noted that the CA–PA–SA/PAN/Al_2_O_3_10 form-stable PCCFMs were hung in the climatic chamber for the accelerated thermal cycling experiment. If the leakage behavior occurred during the measurement, the CA–PA–SA eutectic mixture leaking out of the form-stable PCCFMs would be removed from the membranes and dropped by gravity, which would lead to the decrease of phase change enthalpies. The data listed in Table 2 indicated that no significant change in phase change enthalpies of the CA–PA–SA/PAN/Al_2_O_3_10 form-stable PCCFMs was observed, suggesting that the CA–PA–SA/PAN/Al_2_O_3_10 form-stable PCCFMs had good thermal reliability and reusability in terms of thermal energy storage and release properties.

It is worthwhile to note that the phase change temperatures and enthalpies of form-stable PCCFMs are important thermal performance characteristics that could obviously affect whether they can be used in the specific practical engineering applications. For example, Xu and coworkers reported that the PCMs should have suitable phase change temperature of about 18–30 °C to maintain thermal comfort for building energy-saving applications [29]. Mondal reported that an important performance requirement of a PCM for temperature-regulating fibers and textiles application is that its melting temperature should be between 15 °C and 35 °C [30]. Table 3 summarizes the phase change peak temperatures and enthalpies of the CA–PA–SA/PAN/Al_2_O_3_ form-stable PCCFMs and their comparison with some form-stable PCMs reported in the literatures [9,10,13,14,16,17,20,24]. As shown in Table 3, the solid–liquid PCMs loaded in the form-stable PCMs published in the literatures included pure fatty acids (i.e., stearic acid [13] and lauric acid [16]), paraffin [9,14], fatty acid ester (i.e., glycerol monostearate [17]), fatty acid eutectics (i.e., capric–palmitic acid binary eutectic [10] and capric–myristic–stearic acid ternary eutectic [20]) and polyethylene glycol [24]. It was clearly found from Table 3 that the form-stable PCMs, including paraffin/EP [9], CA–PA/diatomite/EG [10], and CA–MA–SA/CA [20], exhibited similar phase change peak temperatures with those of the CA–PA–SA/PAN/Al_2_O_3_ form-stable PCCFMs reported in this paper (i.e., about 25 °C), but their phase change enthalpies were obviously lower than those of the CA–PA–SA/PAN/Al_2_O_3_ form-stable PCCFMs.

Based on the consideration of phase change temperature, the developed CA–PA–SA/PAN/Al_2_O_3_ form-stable PCCFMs could be considered as promising form-stable PCMs for thermal energy storage applications such as temperature-regulating textiles and building energy conservation.

### 3.3. Thermal Energy Storage and Release Performance

**Figure 5:**
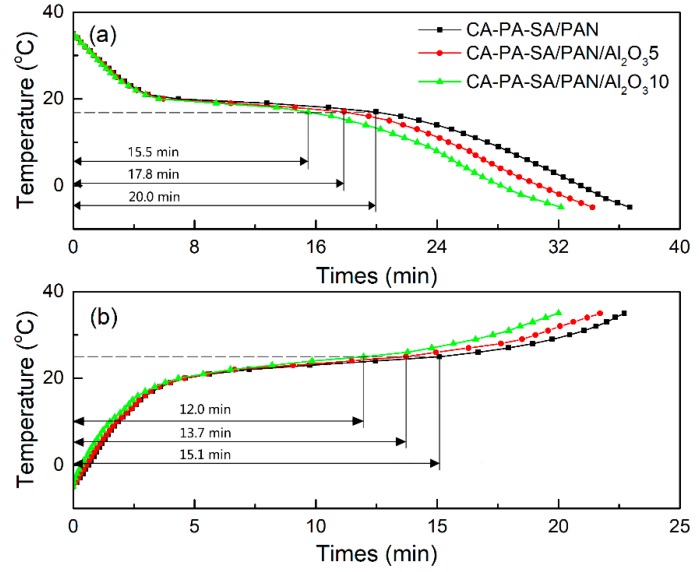
Thermal energy storage and release curves of the CA–PA–SA/PAN/Al_2_O_3_ form-stable PCCFMs with different amounts of Al_2_O_3_: (**a**) thermal energy release, (**b**) thermal energy storage.

The improvement of heat transfer efficiencies of the CA–PA–SA/PAN/Al_2_O_3_ form-stable PCCFMs with different mass fractions of Al_2_O_3_ nanoparticles was discussed through the comparison of melting and freezing times during phase change processes. The corresponding temperature–time curves acquired from thermal performance tests are shown in Figure 5. The interval time between the initial temperature (around −5 °C) and the melting peak temperature (around 25 °C) was defined as the melting time, and the interval time between the initial temperature (around 35 °C) and the freezing peak temperature (around 17 °C) was defined as the freezing time. As shown in Figure 5, the melting/freezing times were determined as about 15.1/20.0 min for CA–PA–SA/PAN, 13.7/17.8 min for CA–PA–SA/PAN/Al_2_O_3_5, and 12.0/15.5 min for CA–PA–SA/PAN/Al_2_O_3_10, respectively. Apparently, the corresponding melting/freezing times were shortened by approximately 9%/11% (CA–PA–SA/PAN/Al_2_O_3_5) and approximately 21%/23% (CA–PA–SA/PAN/Al_2_O_3_10) compared to those of the CA–PA–SA/PAN form-stable PCCFMs. These results illustrated a significant increase in heat transfer rates with the increase of content of the Al_2_O_3_ nanoparticles, which can be attributed to the formation of heat conduction network helping to provide an efficient percolating path for heat flow in the CA–PA–SA/PAN/Al_2_O_3_ form-stable PCCFMs owing to the addition of Al_2_O_3_ nanoparticles.

## 4. Conclusions

The novel CA–PA–SA/PAN/Al_2_O_3_ form-stable PCCFMs were prepared by developing electrospun PAN/Al_2_O_3_ nanofibrous membranes with different mass ratios of Al_2_O_3_ nanoparticles as supporting materials to incorporate CA–PA–SA ternary eutectic via physical adsorption for thermal energy storage. The SEM images revealed that the CA–PA–SA ternary eutectic was successfully absorbed into the three-dimensional porous network structure of electrospun PAN/Al_2_O_3_ nanofibrous membranes owing to the high wetting ability of CA–PA–SA ternary eutectic, the capillary effect, and surface tension force of nanofibrous membranes. The melting peak temperatures and melting enthalpies of the CA–PA–SA/PAN/Al_2_O_3_ form-stable PCCFMs were approximately 25 °C and 131–139 kJ/kg, respectively. The absorption capacities were determined as about 90–95%. The results of thermal performance tests indicated that there was a significant improvement in heat transfer rates of the CA–PA–SA/PAN/Al_2_O_3_ form-stable PCCFMs as the Al_2_O_3_ nanoparticles loading increased. The melting and freezing times of the CA–PA–SA/PAN/Al_2_O_3_10 form-stable PCCFMs were shortened by around 21% and 23%, respectively, in comparison with those of the CA–PA–SA/PAN form-stable PCCFMs. Therefore, based on these results, the developed CA–PA–SA/PAN/Al_2_O_3_ form-stable PCCFMs are concluded to have potential applications in the fields of temperature-regulating fibers and textiles, as well as building energy conservation.

## Figures and Tables

**Table 1:** The peak onset temperatures (*T_o_*), melting peak temperatures (*T_m_*), freezing peak temperatures (*T_f_*), peak end temperatures (*T_e_*), melting enthalpies (*ΔH_m_*), and freezing enthalpies (*ΔH_f_*) of the CA–PA–SA ternary eutectic and CA–PA–SA/PAN/Al_2_O_3_ form-stable PCCFMs with different amounts of Al_2_O_3_ nanoparticles.

Samples	Melting Process				Freezing Process			
	*T_o_* (°C)	*T_m_* (°C)	*T_e_* (°C)	*ΔH_m_* (kJ/kg)	*T_o_* (°C)	*T_f_* (°C)	*T_e_* (°C)	*ΔH_f_* (kJ/kg)
CA–PA–SA	19.72	25.12	33.08	145.7	17.64	15.60	6.29	144.5
CA–PA–SA/PAN	19.10	24.06	27.02	138.6	17.50	17.17	11.30	137.4
CA–PA–SA/PAN/Al_2_O_3_5	19.17	24.29	28.85	132.3	17.33	16.81	9.59	130.3
CA–PA–SA/PAN/Al_2_O_3_10	19.55	25.20	29.56	131.1	17.46	16.55	9.52	127.0

**Table 2:** The peak onset temperatures (*T_o_*), melting peak temperatures (*T_m_*), freezing peak temperatures (*T_f_*), peak end temperatures (*T_e_*), melting enthalpies (*ΔH_m_*), and freezing enthalpies (*ΔH_f_*) of the CA–PA–SA/PAN/Al_2_O_3_10 form-stable PCCFMs after 50 and 100 thermal cycles.

Cycle No.	Melting Process				Freezing Process			
	*T_o_* (°C)	*T_m_* (°C)	*T_e_* (°C)	*ΔH_m_* (kJ/kg)	*T_o_* (°C)	*T_f_* (°C)	*T_e_* (°C)	*ΔH_f_* (kJ/kg)
50 cycles	19.48	27.33	33.61	131.1	18.24	13.76	6.47	129.7
100 cycles	19.47	26.93	33.39	130.6	18.25	13.84	6.51	129.4

**Table 3:** Comparisons on thermal performance data including melting peak temperatures (*T_m_*), freezing peak temperatures (*T_f_*), melting enthalpies (*ΔH_m_*), and freezing enthalpies (*ΔH_f_*) of the CA–PA–SA/PAN/Al_2_O_3_ form-stable PCCFMs with those of some form-stable PCMs reported in the literatures.

Form-Stable PCMs	Melting Process		Freezing Process		References
	*T_m_* (°C)	*ΔH_m_* (kJ/kg)	*T_f_* (°C)	*ΔH_f_* (kJ/kg)	
paraffin/EP	25.10	63.30	-	-	[9]
CA–PA/diatomite/EG	26.69	98.26	21.85	90.03	[10]
SA/aEVT	65.90	146.8	-	-	[13]
paraffin/EVA/EG–CF	45.63	167.4	-	-	[14]
LA/PA6	44.53	70.44	40.67	57.14	[16]
GMS/PET	57.89	66.99	46.65	66.02	[17]
CA–MA–SA/CA	21.80	69.60	14.50	68.80	[20]
PEG/GO/GNP	65.50	177.8	1.70	170.1	[24]
CA–PA–SA/PAN	24.06	138.6	17.17	137.4	Present work
CA–PA–SA/PAN/Al_2_O_3_5	24.29	132.3	16.81	130.3	Present work
CA–PA–SA/PAN/Al_2_O_3_10	25.20	131.1	16.55	127.0	Present work

EP: expanded perlite; CA–PA: capric–palmitic acid binary eutectic; EG: expanded graphite; SA: stearic acid; aEVT: expanded vermiculite/titanium dioxide composite with acid treatment; EVA: ethylene-vinyl acetate; EG–CF: expanded graphite and carbon fiber; LA: lauric acid; PA6: polyamide 6; GMS: glycerol monostearate; PET: polyethylene terephthalate; CA–MA–SA: capric–myristic–stearic acid ternary eutectic; CA: cellulose acetate; PEG: polyethylene glycol; GO: graphene oxide; GNP: graphene nanoplatelets.
